# Perspective: Insights on the Nomenclature of Cytokines and Chemokines

**DOI:** 10.3389/fimmu.2020.00908

**Published:** 2020-05-15

**Authors:** Albert Zlotnik

**Affiliations:** Department of Physiology and Biophysics, University of California, Irvine, Irvine, CA, United States

**Keywords:** cytokines, chemokines, nomenclature, evolution, function

## Summary

The human genome contains some 23,000 genes. Many of these are important in immunology and we have witnessed a very large increase in the characterization of novel genes important in the function of the immune system. Along with these discoveries, issues related to nomenclature have arisen. Often the names proposed for these novel genes and the proteins they encode result in confusion for a new field of research. Here I explain how nomenclature can also help bring important biological insights into the functions of cytokines and chemokines.

## Introduction

Immunology has advanced dramatically in the last 30 years and along with this progress we have witnessed the identification of many novel genes encoding proteins that have important functions in the immune system. Among these are the cytokines, which represent small secreted proteins (10–30 KDa) that are typically produced by cells of the immune system upon activation, and which play pivotal roles in the development and control of immune responses. The history of the cytokines starts in the second half of the 1970's when many groups realized that activated lymphocytes produced secreted proteins that had dramatic effects on other leukocytes. The typical experiment involved the activation of spleen cells with mitogens and the characterization of the biological activities of the supernatants derived thereof. The soluble mediators were given names of the assays that detected their activities like “macrophage activation factor” or “macrophage inhibitory factor.” Several teams started to apply biochemical efforts to distinguish or molecularly characterize the mediators of these activities and this led to the realization that two of the earliest cytokines exhibited specific biochemical characteristics. This led to the identification of the first two interleukins, interleukin 1 and interleukin 2, which were named at the Second International Lymphokine Conference (which was held in Interlaken, Switzerland). Doubtless the venue site inspired the participants to come up with the term “interleukin” which suggests interactions between leukocytes. This example highlights that the issues of nomenclature have been relevant in immunology from the very start of the cytokine field.

Another dramatic step forward was the development of molecular biology tools which led to the initial efforts to “clone” the genes encoding important cytokines. One of the first to be cloned was interferon gamma (by Genentech). At that time biotechnology companies became players in the field and companies like DNAX and Immunex joined the efforts to clone genes of new cytokines. The roster of chemokines by the late 1980s had expanded significantly, up to Interleukin 10 (1). The molecular characterization of these cytokines led in turn to the availability of more molecular tools (Recombinant cytokines, monoclonal antibodies against them), that led to milestone discoveries in immunology like the definition of Th1 and Th2 immune responses (2).

A common belief was that cytokine biology held the key to novel therapeutics. This turned out to be correct, but not as originally conceived. Initial excitement about IL-1 and IL-2 as therapeutics did not yield hoped for breakthroughs. On the other hand, the cytokine field has yielded several very important therapeutics including anti-TNFα antibodies (3), RANKL (4), Erythropoietin or G-CSF (5). The development of these therapeutics has validated the original hopes in the field.

Unfortunately, the cytokine field remains a nomenclature minefield. The interleukins ended up being very difficult to organize. It is still unclear what qualifies a novel cytokine to receive the “interleukin” designation. For example, among the >40 human chemokines (chemotactic cytokines) only one received interleukin designation (interleukin 8). Conversely, there are many interleukins that are related evolutionarily to each other but this is not apparent from their names (IL-4 and IL-13, IL-2, IL-15 and IL-21, IL-10, and IL-22, etc.). In retrospect, the term “interleukin” had a significant advantage: it is a “neutral” designation, one that does not describe a specific characteristic or biological activity. In contrast, consider cytokines like interferon gamma (IFNγ); which is a major immunoregulatory cytokine, and this is what it is known for (not its “interferon” bioactivity). It is a major macrophage activator [including induction of antigen presenting activity (6)]. Thus, this is an example of a cytokine that received a name based on one of the first biological activities detected, even though it is not one of the most relevant (that it would eventually be shown to have).

## Cytokines, Evolution, and Nomenclature

The importance of good nomenclature can be explained by reviewing the experience of naming an important subfamily of cytokines, namely, the chemotactic cytokines or chemokines. As we shall see, the development of a systematic nomenclature for this subfamily lead to important insights into its evolution.

The chemokines represent one of the largest subfamilies of cytokines. There are more than 48 human chemokines described. This family is an excellent example of both nomenclature pitfalls as well as the power of studying a family in the context of its molecular evolution. When the first chemokines were identified, all of them were found to belong to two subclasses: the CXC family (where the first two cysteines were separated by another aminoacid) that tended to attract neutrophils, and the CC family that attracted monocytes and selected T cell subpopulations. Importantly, all the genes encoding CXC chemokines were located in a cluster in human chromosome 4 while the CC chemokines were located in a cluster in human chromosome 17 (7). However, later on other chemokines were identified, and a highly significant one was lymphotactin (now called XCL1) whose encoding gene was located not in any of those clusters but instead in chromosome 1 (8). Subsequently many other chemokines were identified and their genes, like lymphotactin, were located all over the genome (not in the original CXC or CC chemokine clusters).

Now that we know most (if not all) the members of the chemokine superfamily, an interesting evolutionary story has emerged. The chemokines can be subdivided into inflammatory and homeostatic, depending on their expression patterns (homeostatic are expressed without apparent stimuli in selected tissues or organs while the inflammatory typically are expressed during inflammatory conditions). Interestingly, the chemokines whose genes were located in clusters were the inflammatory chemokines, while the genes encoding the homeostatic chemokines were instead located in isolated chromosomal locations away from the clusters. This genomic arrangement can be explained evolutionarily as follows: The oldest and most conserved chemokines are the homeostatic subfamily, and their genes are located in isolated chromosomal locations because of the process through which the chemokine superfamily arose (gene duplication). In this process, a given chemokine gene would undergo duplication, and the resulting offspring genes would be located in the same chromosomal location and their encoded chemokines would bind the same receptor. These “offspring” chemokines would be free to undergo their own individual evolution (as a result of mutations) that would make them valuable to the host and favor its survival. However, if such a process occurred in a chemokine gene with an important function in either homeostasis or development, the chances that the affected organism would survive and pass on this trait to its offspring were not very good. This explains why chemokines with important developmental functions are very well conserved. An excellent example is CXCL12, which is very important during fetal development of various organs (7). In contrast, chemokines of the inflammatory class regularly underwent gene duplication (probably in recent evolutionary times) and therefore their genes are still located in the same location (clusters). Furthermore, the “offspring” genes of these events still bind the same receptors as the original unduplicated precursor. Thus, the evolution of homeostatic chemokines was likely conservative or static while the evolution of inflammatory chemokines was very dynamic. This explains why the inflammatory chemokines tend to share receptors, while the homeostatic chemokines mostly exhibit a single chemokine-receptor relationship (7). The reason most inflammatory chemokines arose was most likely to confer protection from a particular pathogen that a given species may have encountered. For the latter reason, deletion of a particular inflammatory chemokine is unlikely to result in heavily compromised survival of the mutated organism. In humans, this effect is evident in the delta-32 mutation of the CCR5 receptor. Humans affected with this mutation (which results in lack of expression of CCR5) cannot be infected with the AIDS virus (HIV) (9). Conversely, however, individuals carrying the delta 32 CCR5 mutation can be very susceptible to West Nile virus (10).

The conclusion that inflammatory chemokines likely arose recently in evolution is also supported by the observation that they often do not correspond well between species. For example, CXCL8 (Interleukin 8) exists in humans but not in mice (11). This observation can be explained by postulating that CXCL8 arose after the evolutionary separation of the ancestors that gave rise to humans and mice. Following this event, human ancestors have had different “:infectious experiences” than the ancestors of mice. Hence, the inflammatory chemokines present in each species today reflect the “infectious experience” of the ancestors of each species.

This evolutionary model has important implications. For example, the chromosomal location of a particular chemokine can allow us to make predictions about the phenotype of, for example, knockout mice for each chemokine. Knockouts of homeostatic chemokines will likely show a more dramatic phenotype than inflammatory chemokines. Furthermore, if two chemokines share the same chromosomal location, they are likely to share the same receptor (for example, both CCL19 and CCL21 bind CCR7) (11).

Importantly, this evolutionary model is applicable to many superfamilies in the genome and particularly to other cytokines. For example, the genes for IL-4 and IL-13 are located close to each other in human chromosome 5 and their receptors share several features (12).

I can now explain why it was important to talk about gene evolution in immunology in an article focused on nomenclature. The reason is that it was precisely because of nomenclature issues in the chemokine superfamily that we came to understand the evolution of this superfamily. By the year 2000, the nomenclature of the chemokines had become so complicated and confusing that even among experts, the only way to figure out which chemokine we were talking about was to refer back to its sequence. At this point it became obvious that we needed a new standardized nomenclature. The new proposed nomenclature built on the chemokine receptor nomenclature which already existed, but replaced “receptor” for “ligand” (i.e., R for L). Thus, the ligands became CXCL (+ a number) or CCL (+ a number). Luckily, the groups annotating the genome had already allocated numbers to the chemokines but had used a different abbreviation (Small Cytokine subfamily A: SCYA for CC chemokines chemokines or SCYB for CXC chemokines chemokines). Thus, we ended up with CCL21, for example, for a CC chemokine ligand whose gene was originally designated *SCYA21*.

The availability of this new nomenclature allowed experts in the field to produce new figures depicting all the superfamily. Some of these showed the correspondence between receptors and ligands, and the chromosomal locations of the latter. What became immediately apparent was that chemokines whose genes were in the same chromosomal location tended to have the same chemokine receptors; it also became obvious that the genes of the homeostatic chemokines were located throughout the genome while the inflammatory chemokines were in clusters and the latter did not correspond well between species (11). In other words, the new nomenclature allowed us to take a “global view” of this superfamily that fit a compelling evolutionary model for this subfamily of cytokines.

This is therefore a nomenclature story that led to a significant scientific advance. It also underscores the importance of developing a logical nomenclature that has the strong potential to facilitate the study of a particular field.

In the case of the cytokines, there are several superfamilies whose evolution parallel the chemokines. These include the Tumor necrosis factors, the transforming growth factors, and the interferons, among others. I should point out that there are still new cytokines to be identified, if not specifically of importance in immunology, certainly produced in other organs where they likely play an important function. Recently, a study highlighted the fact that most researchers work only on a minority of human genes (13). This situation suggests that there are still many novel genes to be identified and they will need names. We recently identified one of these novel genes (*C17ORF99*) which encodes a novel cytokine we called Interleukin 40 (14).

I think that it is important, when describing a novel gene/molecule, to carefully think about the implications of the name proposed for such a molecule, because it will likely affect the field of research that its discovery will generate. It may be especially important to avoid cheeky or philosophical names. Perhaps a systematic nomenclature that takes structural features or relation to a particular protein family (derived from analyses of characteristics of the encoded protein), where the gene is expressed, rather than its nascent function may be the most likely to prevent a future confusing situation. An estimated 10% of the human genome encodes secreted proteins, and therefore there likely remain many cytokine-like proteins to be described. I hope that these insights may help choose better nomenclature for these proteins.

## Author Contributions

AZ conceived and wrote this opinion on cytokine and chemokine nomenclature based on his experience in the field.

## Conflict of Interest

The author declares that the research was conducted in the absence of any commercial or financial relationships that could be construed as a potential conflict of interest.
